# Relationship between Inflammatory Cytokines and Uric Acid Levels with Adverse Cardiovascular Outcomes in Patients with Stable Coronary Heart Disease

**DOI:** 10.1371/journal.pone.0045907

**Published:** 2012-09-21

**Authors:** Dietrich Rothenbacher, Andrea Kleiner, Wolfgang Koenig, Paola Primatesta, Lutz P. Breitling, Hermann Brenner

**Affiliations:** 1 Institute of Epidemiology and Medical Biometry, Ulm University, Ulm, Germany; 2 Department of Internal Medicine II-Cardiology, University of Ulm Medical Center, Ulm, Germany; 3 Global Clinical Epidemiology, Novartis Pharma AG, Basel, Switzerland; 4 Division of Clinical Epidemiology and Aging Research, German Cancer Research Center, Heidelberg, Germany; Brigham and Women’s Hospital and Harvard Medical School, United States of America

## Abstract

**Background:**

So far it is unclear whether the association between serum uric acid (SUA), inflammatory cytokines and risk of atherosclerosis is causal or an epiphenomenon. The aim of the project is to investigate the independent prognostic relationship of inflammatory markers and SUA levels with adverse cardiovascular outcomes in a patient population with stable coronary heart disease (CHD).

**Methods:**

SUA, C-reactive protein (CRP) and interleukin (IL)-6 were measured at baseline in a cohort of 1,056 patients aged 30–70 years with CHD. Cox proportional hazards model was used to determine the prognostic value of these markers on a combined CVD endpoint during eight year follow-up after adjustment for covariates.

**Results:**

For 1,056 patients with stable coronary heart disease aged 30–70 years (mean age 58.9 years, SD 8.0) follow-up information and serum measurements were complete and n = 151 patients (incidence 21.1 per 1000 patients years) experienced a fatal or non-fatal CVD event during follow-up (p-value = 0.05 for quartiles of SUA, p = 0.002 for quartiles of CRP, p = 0.13 for quartiles of IL-6 in Kaplan-Meier analysis). After adjustment for age, gender and hospital site the hazard ratio (HR) for SUA increased from 1.37 to 1.65 and 2.27 in the second, third, and top quartile, when compared to the bottom one (p for trend <0.0005). The HR for CRP increased from 0.85 to 0.98 and 1.64 in the respective quartiles (p for trend 0.02). After further adjustment for covariates SUA still showed a clear statistically significant relationship with the outcome (p for trend 0.045), whereas CRP did not (p for trend 0.10).

**Conclusion:**

The data suggest that compared to inflammatory markers such as CRP and IL-6 serum uric acid levels may predict future CVD risk in patients with stable CHD with a risk increase even at levels considered normal.

## Introduction

Low grade inflammation plays a major role in coronary heart disease (CHD) and especially C-reactive protein (CRP) and interleukin-6 (IL-6, a major pro-inflammatory cytokine) are relevant molecules in this process [1]. CRP is primarily synthesized in the liver via IL-6-dependent biosynthesis, shows a long-term stability during long-term storage at −80°Celsius, and its good analytical properties have enabled the development of a very extensive and robust database for the association of CRP with future cardiovascular disease (CVD) outcomes. According to a meta-analysis of the Emerging Risk Factors Collaboration including 160,309 subjects CRP shows a positive association with risk of cardiovascular disease similar to non-high density lipoproteins or systolic blood pressure [2]. However, its added value as a prognostic tool over and above traditional cardiovascular risk factors such as systolic blood pressure and lipid values is still debated.

A relationship between hyperuricemia or, gout and CVD has been described in many studies [3], [4]. Acute gout (for which a raised serum uric acid (SUA) is the most important single risk factor) is an inflammatory arthritis induced by deposition of monosodium urate crystals, predominantly in the joints of the lower limbs [5]. Multiple cytokines and chemokines such as interleukin (IL)-1β and IL-6 are involved in this inflammatory process [6]. As SUA has also direct immune-modulating effects [7], a direct involvement of SUA in the inflammatory cascade associated with atherosclerosis seems possible. SUA as well as gout are also related to many cardiometabolic risk factors such as obesity and the metabolic syndrome, hypertension, diabetes, renal disease, and other inflammatory processes. Notably, CVDs are the greatest threat for patients with gout and hyperuricemia [8]. However, a simultaneous investigation of serum urate acid levels and inflammatory parameters considering a large number of potentially related factors such as renal function and established cardiovascular risk factors to further disentangle the causality of these interrelated factors in a long-term observational study is lacking.

The aim of the present project is to investigate the relationship of inflammatory markers with serum uric acid level in a patients population with stable coronary heart disease (CHD) at baseline and also investigate the independent prognostic relationship of inflammatory markers and serum uric acid levels for subsequent fatal-and non-fatal CVD events under special consideration of potential confounding factors such as markers of cardiac and renal dysfunction and hypertension.

## Materials and Methods

### Study Population

All patients with CHD (International Classification of Diseases, 9th Rev. codes. 410–414) aged 30–70 years and participating in an in-hospital rehabilitation program between January 1999 and May 2000 in two cooperating hospitals (Schwabenland-Klinik, Isny and Klinik im Südpark, Bad Nauheim, Germany) were eligible for enrolment in the study (initial response 58%), further details can be found elsewhere [9]. In Germany, all patients after an acute coronary syndrome (ACS) or elective coronary artery bypass grafting (CABG) are offered a comprehensive in-hospital rehabilitation program after discharge from the acute care hospital. The aim of this 3-weeks program is the reduction of cardiovascular risk factors, improvement of health related quality of life, and the preservation of the ability to work (the latter only if a patient was still at work at the onset of disease, otherwise the prevention of nursing care). This in-hospital rehabilitation program usually starts approximately three weeks after the acute event or CABG when patients are in stable conditions (i.e. free of angina). In the current study, only patients who were admitted within three months after the acute event or CABG were included.

All subjects gave written informed consent and the study protocol conforms to the ethical guidelines of the 1975 Declaration of Helsinki as reflected in a *priori* approval by the institution’s human research committee. The study was approved by the Ethics Boards of the Universities of Ulm and Heidelberg and of the Physicians’ chamber of the States of Baden-Wuerttemberg and Hessen (Germany).

### Data Collection

At the beginning of the in-hospital rehabilitation program all patients filled out a standardized questionnaire containing socio-demographic information and medical history (including history of gout and year of first diagnosis by a physician). In addition, information was taken from the patients’ hospital charts (current hospitalization). In all patients active follow-up was conducted 1, 3, 4.5, 6 and 8 years after discharge from the rehabilitation centre. Information was obtained from the patients using a mailed standardized questionnaire. Information regarding subsequent CVD events and treatment since discharge from the in-hospital rehabilitation clinic was obtained from the primary care physicians also by means of a standardized questionnaire. If a subject had died during follow-up, the death certificate was obtained from the local Public Health Department and the main cause of death was coded according to the International Classification of Diseases (ICD-9 pos. 390–459: ICD-10 pos. I0–I99 and R57.0). Subsequent CVD events were defined either as CVD as the main cause of death (as stated in the death certificate), non-fatal myocardial infarction (MI), or non-fatal ischemic stroke. All non-fatal subsequent events were reported by the primary care physicians.

### Laboratory Measurements

High-sensitive (hs)-CRP concentrations were measured by immunonephelometry on a Behring Nephelometer II (N Latex CRP mono Dade-Behring, Marburg). Cystatin C was determined on the same device (Dade-Behring, Marburg). Interleukin-6 was measured with a high-sensitivity ELISA (R&D Systems). NT-proBNP was measured by electrochemiluminescence on an Elecsys 170 (Roche Diagnostics, Mannheim, Germany), and lipoprotein-associated phospholipase A_2_ (Lp-PLA_2_) mass was measured by ELISA (diaDerxus Co. South San Francisco, USA). High-sensitivity (hs)-troponin measurements were done on an Elecsys 2010 platform (Roche Diagnostics, Penzberg, Germany). Serum uric acid levels, creatinine, blood lipids and leukocyte count were done by routine methods in both participating hospitals. All markers were measured in a blinded fashion. Chronic kidney disease (CKD) was defined according to the Chronic Kidney Disease Epidemiology Collaboration (CKD-EPI) creatinine-based equation including also age, gender and ethnicity [10]. CKD was defined as eGFR of less than 60 mL/min/1.73 m^2^.

### Statistical Analysis

The study population and the subpopulation with a self-reported history of gout were described with respect to various sociodemographic and medical characteristics. The relation of uric acid values (distribution was categorized with gender-specific quartiles) and inflammatory markers (CRP, IL-6) with non-fatal and fatal CVD events during follow-up was assessed by the Kaplan-Meier and life table method and quantified by means of the log-rank test. Then the Cox proportional hazards model was employed to assess the independent association of these markers with the risk of mortality and of subsequent CVD events (hazard ratios, HR, and their 95% confidence intervals, CI).

A basic model (model 1) was adjusted for age (years), gender, and hospital site. In a second step the following potential confounders were additionally considered for multivariable analyses: body mass index (BMI, kg/m^2^), smoking status (never, current, ex-smoker), alcohol consumption (abstainers,<125 g, and ≥125 g ethanol per week), duration of school education (<10 years, ≥10 years), family status (married, other), history of myocardial infarction (yes, no), history of hypertension (yes, no), history of diabetes mellitus (yes, no), severity of CHD (number of affected epicardial vessels at baseline), initial management of CHD (conservative, percutaneous coronary intervention, PCI, CABG), chronic kidney disease (yes, no), intake of allopurinol (yes, no), intake of ß-blockers (yes, no), intake of ACE-inhibitors (yes, no), intake of diuretics (yes, no), intake of lipid-lowering drugs (yes, no), HDL-cholesterol (mg/dL), LDL-cholesterol (mg/dL) (model 2). Only those variables were added to the model which were significant predictors of a subsequent event at an α-level of 0.1 or which changed the parameter estimates for the main variables by more than 10%. To further explore the so far yet unclear role of renal, the potential of effect modification between renal function (glomerular filtration rate as estimated by means of CKD-EPI) and SUA was also done by including a product term to the model.

Measures of model fit, discrimination, reclassification and calibration were assessed with Cox proportional hazards regression. The net reclassification improvement (NRI) by adding SUA was calculated according to the risk strata of 5%, 10% and >20% of predicted probability for a cardiovascular event. Furthermore, the integrated discrimination improvement (IDI) was assessed which estimates the extended model’s improvement in the difference in predicted probabilities [11], [12]. All statistical procedures were carried out with the SAS statistical software package (release 8.2, Cary, NC (USA), SAS Institute Inc. 1999).

## Results

Overall, 1,206 patients with a diagnosis of CHD within the past 3 months were included at baseline during the in-hospital rehabilitation program. Eight-year follow-up information and serum measurements were complete for 1,056 patients (87.6%). The main characteristics of the overall study population and the sub-population with history of gout are shown in **Table 1**. The mean age of the study population at baseline was 58.9 years, most of the patients were male (84.9%). A history of myocardial infarction or heart failure was reported by 58.3% and 12.5%, respectively. Three-vessel disease was present in 42.6% of the CHD patients at baseline. Median hs-CRP was 3.5 mg/L, median IL-6 was 3.5 pg/mL, and mean serum uric acid was 6.1 mg/dL. In general, patients with a history of gout had a less favourable cardiometabolic risk profile, for example median hs-CRP was 5.0 mg/L, IL-6 was 3.8 pg/mL, and mean serum uric acid level was 6.9 mg/dL. This was also true for renal function and NT-proBNP, as well as for hs-troponin. Duration of history of gout was > five years in 59.7% (n = 142) and between 2 and five years for 18.9% (n = 45) of patients with gout.

**Table 1:** Sociodemographic, Clinical, and Laboratory Characteristics in Patients With Clinically Manifest Coronary Heart Disease and in the Sub-population with History of Gout.

Characteristics at Baseline	All patients with CHD	Sub-population without history of gout at baseline	Sub-population with history of gout at baseline	P-Value^C^
N	1056	806	229	
Age (years) (x?, SD)	58.9 (8.0)	58.3 (8.3)	60.8 (6.4)	0.0002
Men, n (%)	897 (84.9 %)	678 (84.1%)	205 (89.5 %)	0.04
History of myocardial infarction, n (%)	616 (58.3 %)	487 (60.4%)	116 (50.7 %)	0.008
History of heart failure, n (%)	128 (12.5 %)	95 (12.0%)	32 (14.5 %)	0.32
Clinical score (angiographic evaluation)				
- 1 vessel disease	258 (24.4 %)	201 (24.9%)	54 (23.6 %)	
- 2 vessel disease	282 (26.7%)	209 (25.9%)	66 (28.8 %)	
- 3 vessel disease	450 (42.6 %)	341 (42.3%)	101 (44.1 %)	
- unknown	51 (4.8 %)	42 (5.2%)	6 (2.6 %)	0.72
School education <10 yr, n (%)	629 (59.6 %)	481 (59.7%)	134 (58.5 %)	0.75
Smoking status				
Never	333 (31.5 %)	262 (32.5%)	68 (29.7 %)	
Former	671 (63.5 %)	504 (62.5%)	150 (65.5 %)	
Current	52 (4.9 %)	40 (5.0%)	11 (4.8 %)	0.70
Alcohol consumption				
abstainers	267 (25.4%)	211 (26.3%)	48 (21.2%)	
<125 g/week	418 (39.8%)	338 (42.1%)	74 (32.6%)	
125+g/week	365 (34.8%)	253 (31.6%)	105 (46.3%)	0.0002
Body mass index (kg/m^2^), (x?, SD)	27.1 (3.5)	26.9 (3.5)	27.7 (3.5)	0.0032
Body mass index >30 kg/m^2^ (n, %)	188 (17.8%)	129 (16.0%)	53 (23.1%)	0.013
History of diabetes, n (%)	182 (17.2 %)	133 (16.5%)	43 (18.8 %)	0.42
Total cholesterol (mg/dl) (x?, SD)	169.0 (32.4)	169.3 (33.0)	168.2 (30.3)	0.69
LDL-cholesterol (mg/dl) (x?, SD)	100.6 (29.0)	101.0 (29.3)	99.5 (28.2)	0.59
HDL-cholesterol (mg/dl) (x?, SD)	39.4 (10.5)	39.8 (10.8)	37.9 (9.2)	0.04
C-reactive protein (mg/L)^a^	3.5 (1.2; 8.4)	3.1 (1.2;8.0)	5.0 (1.6; 10.0)	0.003
Interleukin-6 (pg/mL)^a^	3.5 (2.2; 7.1)	3.4 (2.2;6.8)	3.8(2.2; 8.0)	0.098
Serum uric acid (mg/dL)( x?, SD)	6.1 (1.7)	5.9 (1.6)	6.9 (1.8)	<0.0001
*Cont. table1*				
Creatinine Clearance (mL/min)^a^	93.4 (78.1; 114.9)	95.6 (79.0;115.9)	87.1 (76.5; 106.3)	0.0006
Cystatin C (mg/L)^a^	1.0 (0.9; 1.2)	1.0 (0.9;1.2)	1.1 (1.0; 1.3)	<0.0001
Estimated GFR (CKD-Epi)a, b	85.7 (73.1; 96.7)	88.0 (74.7;97.8)	78.6 (67.5; 91.2)	<0.0001
NT-proBNP (pg/mL)^a^	565.2 (277.9; 1101.0)	537.0 (274.4;1073.0)	656.4 (279.6; 1145.0)	0.17
Hs-troponin (ng/L)^a^	10.8 (5.1; 18.7)	10.2 (5.0;18.3)	12.1 (5.6; 21.4)	0.04

a median, 25^th^ and 75^th^ percentile cut-point.

b CKD-Epi = Chronic Kidney Disease Epidemiology Collaboration equation.

c based on Wilcoxon Two-Sample Test for continuous variables, Chi-Square Test for proportions.

**Figure 1:**
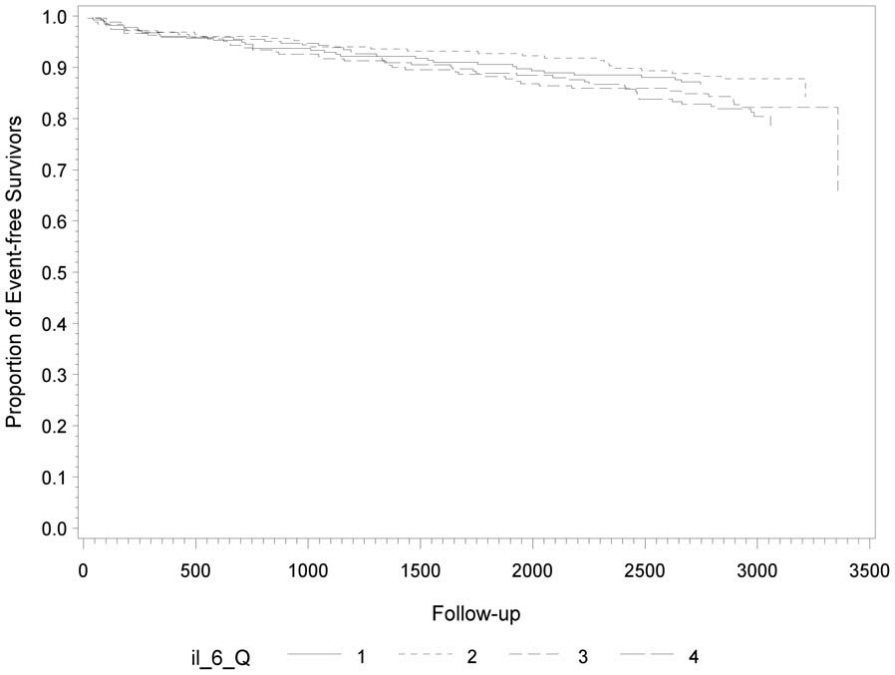
Kaplan-Meier Estimates of Subsequent Fatal and Non-Fatal CVD Events) during Follow-Up According to Quartiles of Uric Acid at Baseline.

**Figure 2:**
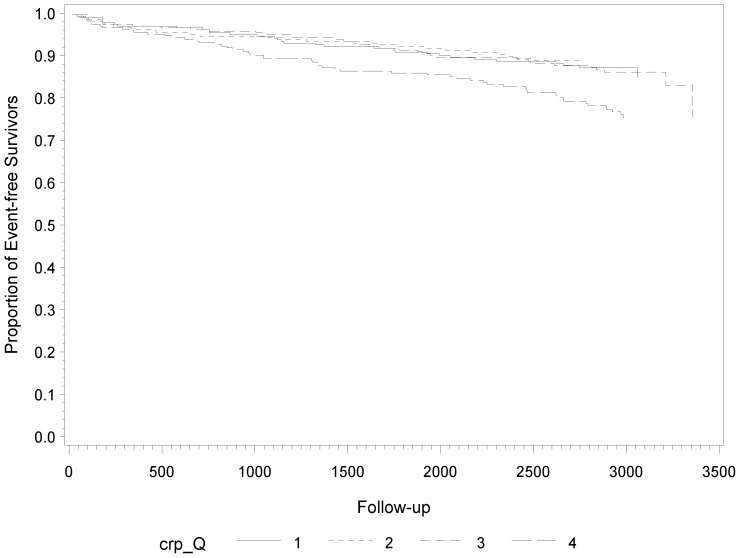
Kaplan-Meier Estimates of Subsequent Fatal and Non-Fatal CVD Events during Follow-Up According to Quartiles of CRP at Baseline.

**Figure 3:**
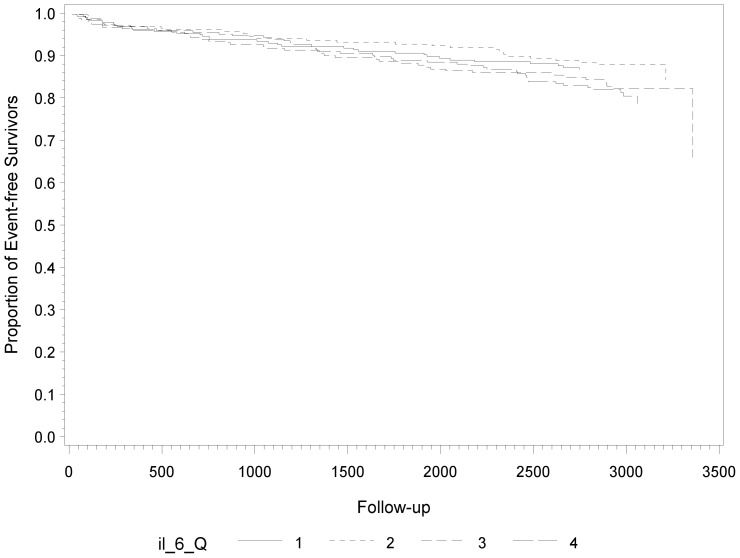
Kaplan-Meier Estimates of Subsequent Fatal and Non-Fatal CVD Events During Follow-Up According to Quartiles of IL-6 at Baseline.

**Figures 1** **to** **3** show the Kaplan-Meier plots and incidence rates by SUA, CRP, and IL-6 levels of subsequent fatal and non-fatal CVD events. There was a clear increase of the incidence of fatal and non-fatal CVD events with increasing SUA levels during follow-up (p- = 0.05) (Figure 1). The incidence in the bottom quartile was 15.6 events per 1000 patient years and increased to 28.9 per 1000 patient years in the top quartile. The results for CRP were quite even stronger (p = 0.002) and the incidence in the bottom quartile and the top quartile was 17.6 and 33.1 per 1000 patient years, respectively **(** **Figure 2** **)**. We found no statistically significant association between IL-6 levels and subsequent fatal and non-fatal CVD events (p = 0.13) (**Figure 3**).

**Table 2** shows the results of the multivariable analysis after adjustment for potential confounding factors. In model 1 (adjusted for age, gender and hospital site) the hazard ratio (HR) for SUA increased from 1.37 to 1.65 and 2.27 in the second, third, and top quartile, when compared to the bottom one after adjustment for age, gender and hospital site (p for trend = 0.0005). The HR for CRP increased from 0.85 to 0.98 and 1.64 in the respective quartiles (p for trend 0.02). After further adjustment for covariates in model 2, SUA still showed a clear statistically significant relationship with the outcome (p = 0.045), whereas CRP did not (p for trend = 0.10). If this analysis was restricted to men, the respective HR were 1.20 (95% CI 0.68–2.10), 1.51 (95% CI 0.86–2.65), and 1.81 (95% CI 1.02–3.20) when the second, third and fourth SUA quartile were compared to the bottom on (p for trend = 0.03). The respective values for CRP were 0.91 (95% CI 0.51–1.64), 0.86 (95% CI 0.47–1.56), and 1.48 (0.85–2.56) (p for trend = 0.12).

**Table 2:** Association of Uric Acid Concentrations and hs-CRP (categories, e.g. quartiles) at Baseline With Subsequent Fatal and Non-Fatal CVD Events During Follow-Up.

	Results of Multivariate Analysis		
	Model 1 Hazard Ratio (95% CI) adjusted for age, gender and hospital site	Model 2 Hazard Ratio (95% CI) adjusted for multiple covariates^a^	*Model 3 Hazard Ratio (95% CI) adjusted for multiple covariates* b
Uric Acid			
Bottom Quartile	1 referent	1 referent	*1 referent*
Second	1.37 (0.83–2.23)	1.14 (0.67–1.93)	*1.17 (0.68–1.99)*
Third	1.65 (1.01–2.70)	1.51 (0.90–2.54)	*1.49 (0.88–2.53)*
Top Quartile	2.27 (1.41–3.66)	1.61 (0.95–2.73)	*1.49 (0.85–2.61)*
	p for trend 0.0005	p for trend 0.045	*p for trend 0.11*
Hs-CRP			
Bottom Quartile	1 referent	1 referent	*1 referent*
Second	0.85 (0.51–1.41)	0.65 (0.37–1.15)	*0.66 (0.37–1.16)*
Third	0.98 (0.60–1.59)	0.68 (0.39–1.18)	*0.67 (0.38–1.17)*
Top Quartile	1.64 (1.05–2.56)	1.33 (0.81–2.20)	*1.27 (0.76–2.10)*
	p for trend 0.02	p for trend 0.10	*p for trend 0.17*

a considered main model - beside the main factors (the variables age, gender and hospital site), the following potential confounders were included in multivariable analyses: smoking status (never, current, ex-smoker), history of myocardial infarction (yes, no), history of diabetes mellitus (yes, no), severity of CHD (number of affected epicardial vessels at baseline), intake of ACE-inhibitors (yes, no), intake of allopurinol (yes, no), HDL-cholesterol (mg/dl), LDL-cholesterol (mg/dl). Only those variables were added to the model which were significant predictors of a subsequent event at a α-level of 0.1 or which changed the parameter estimates for the main variables by more than 10% *(details see methods for variable selection).*

b adjusted for above listed variables (^a^) and CKD-EPI.

The further explorative adjustment for chronic kidney disease by means of CKD-EPI (which did not qualify to the main model 2, considered our main model) in model 3 decreased the HRs for SUA and CRP. However, the included marker of renal dysfunction may point to an underlying pathophysiological pathway (e.g. chronic kidney disease increases SUA) and the model therefore may already be considered over-adjusted). A history of gout was not associated with the outcome if included in the model in addition to SUA levels.

If SUA and CRP were considered simultaneously in model 2, which was considered the main model, only SUA showed an tentatively increased risk (e.g. the HR of the respective quartile were 1.17, 1.48, and 1.51, when the second, third, and top quartile were compared to the bottom one (p for trend = 0.07); whereas CRP showed no statistically significant association with the outcome (the HRs for CRP were 0.65, 0.68, and 1.28 (p for trend = 0.15) respectively). We did not find any evidence of interaction of sex, age, adiposity, and chronic kidney disease with SUA or CRP.

**Table 3** finally shows various measures of model accuracy with and without SUA. In the main model 2, the addition of SUA improved the model fit. C-statistics demonstrated a very small increase in the area under the curve (from 0.680 to 0.681). The integrated discrimination improvement (IDI) was 0.005 (p = 0.03) and net reclassification improvement (NRI) was 9.9% (p = 0.03) when SUA was added to the model.

**Table 3:** Measures of Model Accuracy With and Without Serum Uric Acid (SUA).

	Basic model^a^	Basic model plus SUA
**Model fit**		
Likelihood ratio (LR)	47.25 (df = 11, p<0.0001)	53.25 (df = 14, p<0.0001)
Akaike’s information criterion (AIC)	1661.8	1661.8
Bayesian information criterion (BIC)	1693.1	1701.6
**Discrimination**		
C-statistic (AUC, 95% CI)	0.680 (0.636–0.724)	0.681 (0.637–0.726)
Integrated discrimination improvement (IDI)**Calibration**		0.005 (p = 0.03)
Net reclassification improvement (NRI)		9.9% (p = 0.03)
Subjects with CVD-event	n_up_/n_down_	20/8
Subjects without CVD-event	n_up_/n_down_	93/97

a adjusted for age, gender and hospital site, smoking status (never, current, ex-smoker), history of myocardial infarction (yes, no), history of diabetes mellitus (yes, no), severity of CHD (number of affected epicardial vessels at baseline), intake of ACE-inhibitors (yes, no), intake of allopurinol (yes, no), HDL-cholesterol (mg/dl), LDL-cholesterol (mg/dl) (*model 2 of* *table 2* *according to variable selection criteria*).

## Discussion

In this study in a high risk group of patients with stable CHD at baseline and long-term follow-up we found a statistically significant relationship between serum uric acid levels and subsequent CVD events that persisted after adjustment for established risk factors. In contrast, this was not the case for CRP and IL-6. Furthermore, the relationship showed a dose-response pattern and notably, a risk increase was already evident within values considered to be in the normal range. The data are in line with the hypothesis that serum acid levels (in contrast to CRP and IL-6) may contribute independently to the pathophysiology of CVD-events in patients with stable CHD.

### Serum Uric Acid and CVD

Gout, the clinical manifestation of hyperuricemia, is the most common cause of inflammatory arthritis, affecting about 1 to 2% of the general population in the Western world. In a representative study conducted within the UK the incidence in 50 to 59 year old was 4.81 and 1.06 per 1000 person years in males and females, respectively, and increased steeply with increasing age [13]. In the latter study it was also evident that gout and CVD comorbidity are closely associated. However, the question whether hyperuricemia, gout, and CVD share only common risk factors such as obesity, diabetes, inflammation, or dyslipidemia, or whether their association is of causal nature is not clear yet and hotly debated [3], [4].

A systematic review and meta-analysis reported an overall modest increase for CHD incidence and mortality, independent of traditional risk factors [14] and a similar result was reported for stroke [15].The finding that the association of uric acid levels and CVD is not only observed in patients with clinical manifestation of gout or hyperuricemia, but also with levels that are considered to be in the normal range [4] is supported by the findings of our study. A large-scale epidemiology study in 28.613 elderly women with 21-year follow-up showed also an independent association of SUA levels on all major forms of CHD mortality including chronic heart disease heart failure, and stroke [16] and risk also increased at SUA levels considered normal. Hyperuricemia has also been described as independent predictor of micro- and macroangiopathies in patients with diabetes [17].

So far in genetic studies, which could overcome issues of confounding and interrelated risk factors, only a causal relationship between uric acid levels and gout has been reported, but no evidence was reported for CHD by means of Mendelian randomization [18]. A recent historical cohort study including 148,217 subjects of a private health insurance company in contrast suggested that increased SUA were associated with graded increase in cardiovascular hospitalization rates independent of established risk factors and severity of kidney function [19]. A large study from Taiwan including 128,569 adults showed similar results for ischemic heart disease not only in the general population, but also in subjects without any risk factor for cardiometabolic syndrome [20]. Animal models suggest a potential causal pathway related to increased left ventricular dysfunction and enhanced myocardial hypertrophy and fibrosis [21].

As uric acid is mainly eliminated by renal excretion, CKD may increase the associated risk. A study in patients with heart failure, however, found that hyperuricemia was especially associated with adverse outcomes in patients without kidney disease and suggested that its mechanistic action should in this case mainly acting via an increased production (and thus be a marker of an increased xanthine oxidase activity) rather than by an intrinsic effect by SUA itself [22]. In our study, including patients with stable CHD, patients with CKD showed a tentatively higher risk of subsequent CVD events compared to patients without CKD, supporting the idea of an intrinsic effect of SUA itself (unfortunately a detailed analysis was not possible due to the low number of patients with CKD (prevalence at baseline was only 9.1%) and CKD did not qualify to our main model).

### Inflammation and CVD

CRP is among the most studied proinflammatory proteins and even trace levels can be reliably measured among healthy subjects. Besides its production in the liver under the transcriptional control of mainly IL-6, it may also be produced locally in vascular smooth muscle cells and in macrophages of atherosclerotic lesions [1]. IL-6 induces a prothromobotic state and has important direct proatherogenic properties in addition to its role in the amplification of a proinflammatory state by a prominent stimulus of production of acute phase proteins like CRP. There is robust evidence that CRP concentration is consistently associated with subsequent CVD, and the results basically are similar to findings reported for other downstream markers of inflammation [2]. However, whether these findings demonstrate a causal association or are modulated by other factors, possibly related to the inflammatory cascade or other risk factors, is unclear yet. In Mendelian randomization studies, which can potentially overcome these issues of confounding as genes are independent from these concurrent risk factors, further hints can be found for a causal relationship. However, although CRP may be considered a risk marker, a causal relationship of CRP with CVD outcomes is rather unlikely based on data from several Mendelian randomization studies [23]–[25].

### Clinical Implications

As more and more evidence is accumulating that the association of SUA and CVD may be causal, supported by mechanistic studies of potential pathways, a clinical trial finally may be warranted to prove or disprove the suggested causal relationship between SUA levels and the risk of subsequent CVD [3], [7]. However, at present there are not yet sufficient data to recommend treatment of asymptomatic hyperuricemia in order to prevent CVD events [4].

### Strength and Limitations

Our study has several strengths as well as limitations that need to be addressed. Firstly, our study included a large group of patients with stable CHD from clinical routine. As the acute events leading to diagnosis of CHD or MI had occurred at least 3 weeks before inclusion in this study, selection of patients with a better prognosis compared to a patient population within the early phase of a newly diagnosed CHD must be assumed. Although we had a large sample of patients with CHD (over 50% with a history of MI), fatal CVD events were limited in this study population. This is explained by the fact that mortality of MI is highest during the pre- and early in-hospital phase. Furthermore, not all patients were willing or able to participate in an in-hospital rehabilitation program. This may provide a further explanation for the underrepresentation of severely ill patients in our study sample. However, it does not explain the consistent and positive association between SUA levels and CVD events.

### Conclusions

The data are in line with the suggestion that serum uric acid levels (in contrast to CRP and IL-6) predict future CVD risk in patients with stable CHD independent of other established risk factors with a risk increase appearing even at SUA levels considered normal. Future studies should investigate whether SUA may contribute independently to the pathophysiology of CVD-events in patients with stable CHD. If corroborated SUA may be considered a target for early prevention.
